# Dendrimers branch out to support mucosal integrity

**DOI:** 10.1002/emmm.201201668

**Published:** 2012-08-22

**Authors:** Dermot Kelleher

**Affiliations:** Department of Clinical Medicine, Institute of Molecular Medicine, Trinity College DublinDublin, Ireland

**Keywords:** dendrimer, diarrhoea, interleukin-6, nanobiotechnology, shigellosis

See related article in EMBO Molecular Medicine http://dx.doi.org/10.1002/emmm.201201290

Dendritic polymer technology represents one of the most exciting areas at the interface of polymer chemistry and medical science. Dendritic polymers or dendrimers are symmetrical structurally perfect branching molecules synthesized from a central core molecule set, which are classified by the number of branching cycles or generations (Walter & Malkoch, 2012). Dendrimers typically contain a central cavity and beyond three generations, they adopt a spherical configuration. An increasing number of branching generations augment the number of functionalizable groups on the surface available to carry specific molecules or to directly interact with reaction partners. Dendritic polymers may have a range of functional properties including the capacity to function as catalysts, as biosensors or even as anti-bacterial agents.

Within biomedicine, dendrimers have been utilized as delivery systems for drugs and in particular hydrophobic agents, radioligands and other imaging molecules. Current research has also focused on dendrimers as vehicles for delivery of DNA with potential for gene therapy. They may be functionalized in a number of ways to improve cellular access through modification or coating of the dendrimer surface. In addition, dendrimers may have similar structural properties to molecules such as hemoglobin, porphyrins and others and have the potential to be modified to produce synthetic variants of naturally occurring biological molecules such as heme to be used as carriers for heme-mimetics as potential blood substitutes (Palivan et al, 2012; Walter & Malkoch, 2012).

» …These studies demonstrate that it is possible to generate synthetic dendrimers capable of interfering with specific residues on innate immune molecules with very profound downstream effects.«

The recent work by Teo et al in EMBO Molecular Medicine (Teo et al, 2012) has identified a further potential application for a surface-modified dendrimer. These investigators identified a key interaction within the innate immune system as the target for the development of specific bioactive dendrimers. Bacteria, which contain lipopolysaccharide (LPS), interact through LPS with a key molecule of the innate immune system, namely TLR4. This molecule, through further interactions with a molecule called MD-2 and a set of downstream signalling events, activates a plethora of immune and inflammatory responses many of which are mediated through a set of interacting molecules such as MyD88, MAL, TRIF and transcription factors including NF-kB and IRFs (Kenny & O'Neill, 2008). Hence, the LPS–TLR4 interaction is regarded as the key interaction between Gram-negative bacteria and the innate immune system with consequences not simply for the elimination of the invading organism or organisms but also for the morbidity induced by the resulting inflammation. Further evidence indicates that other organisms with catastrophic gastrointestinal effects such as *Clostridium difficile*, which do not express LPS, also interact with this system through their surface layer proteins (SLP; Ryan et al, 2011).

**Figure 1:**
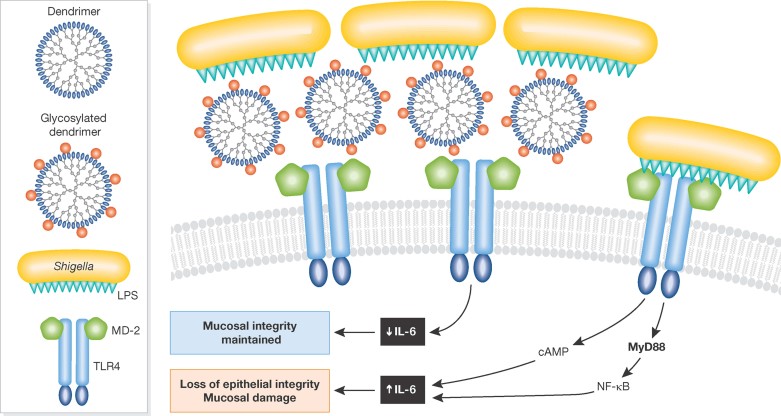
Glycosylated dendrimers bind MD-2 and prevent mucosal damage Interaction of LPS on the surface of intestinal bacteria such as *E. coli* or *Shigella* with the TLR–MD-2 complex on host inflammatory and epithelial cells triggers the production of IL-6 through both NF-κB and cAMP mediated pathways with consequences for mucosal integrity and barrier function. Glycosylated dendrimer molecules can selectively interfere with this interaction with consequent preservation of mucosal integrity.

Building on prior data, which demonstrated biological effects of glycosylated dendrimers and on modelling data demonstrating the potential for glycosylated dendrimer interactions with MD-2 in the TLR4–MD-2–LPS receptor complex, the authors initially generated a glycosylated version of the commercially available polyamidoamine (PAMAM) dendrimer and utilized this molecule in *in vitro* and *in vivo* studies on *Escherichia coli* and *Shigella* signalling through TLR4. While the dendrimer did not have any direct anti-bacterial effects, it substantially reduced monocyte secretion of IL-6, with further reductions in the production of other cytokines such as IL-8, TNF-α and IL-1β. Having demonstrated biological activity, competition studies strongly suggested that the DG molecule was competing with the Lipid A component of LPS for binding to MD-2. These data suggested the possibility that such a molecule could have beneficial biological effects in animal models of enteric infection. The authors then synthesized a substantially smaller but scaleable dendrimer molecule, namely the polypropyletherimine (PETIM)-dendrimer glucosamine (DG), which exhibited similar abilities to compete with Lipid A binding to MD-2 and potent biological activity in a rabbit model of *Shigella* infection. Specifically, in this model, PETIM-DG not only inhibited IL-6 and IL-8 production in rabbits infected with *Shigella* but also dramatically attenuated intestinal damage (Fig 1).

» …This study raises the intriguing possibility that such dendrimers may have a clinical utility in the management of the mucosal damage produced by both gastrointestinal infection and inflammation.«

These studies demonstrate that it is possible to generate synthetic dendrimers capable of interfering with specific residues on innate immune molecules with very profound downstream effects. The therapeutic implications for such research are potentially exciting with regard to treatment of gastrointestinal infectious and inflammatory diseases. Antibiotic resistance is a rapidly increasing problem, which is compounded by indiscriminate antibiotic use at a global level. Such treatment may have unpredictable effects including the development of complications such as pseudomembranous colitis resulting from super-infection with *C. difficile*, an organism also known to interact directly with the innate immune system albeit not through LPS. In addition, the normal homeostasis of gut epithelium with respect to its intestinal flora may be dependent on the interaction of bacterial ligands with mucosal innate immune receptors. Modifications of such interactions either by altering the intestinal flora or by knocking out innate molecules such as *MyD88* can lead to intestinal inflammation and intramucosal haemorrhage (Rakoff-Nahoum et al, 2004). The balance between gut flora and innate immunity may have consequences for inflammatory bowel diseases where, for example mutations in innate immune molecules have been identified in Crohn's disease (reviewed in Abraham & Medzhitov, 2011). Hence, the data reported by Teo et al could have potentially important consequences. Firstly, the capacity to prevent intestinal damage without altering the gut flora in the treatment of gastrointestinal pathogens is an intriguing one and one which deserves further study in the light of the data published in this issue. Such an approach has potential implications for therapy of *E. coli*, *Shigella* and *C. difficile* all of which have been shown to act at least in part through the TLR4 receptor although the full scope of the interactions between SLP and TLR4 in *C. difficile* remains to be defined (Ryan et al, 2011). What may be of particular interest is the potential for such dendrimer-based technologies to play a role in resetting the homeostasis of the gastrointestinal immune system in situations where there has been a breakdown of tolerance or of gastrointestinal barrier function such as in post-infectious diarrhea and possibly inflammatory bowel disease.

It is particularly interesting that modulation of IL-6 secretion is suggested as a possible mechanism for the reduced intestinal damage seen following DG treatment in the *Shigella* rabbit model. IL-6 secretion is triggered through LPS–TLR4 signalling and may involve a rapid cyclic AMP-regulated pathway in addition to the conventional NF-κB mediated signalling events (Song et al, 2007). Of note in murine models, IL-6 or IL-6R blockade substantially ameliorates the severity of colitis (Yamamoto et al, 2000). Moreover, the functional integrity of the intestinal epithelial barrier in response to insults such as haemorrhagic shock is preserved in the IL-6 knockout mouse (Yang et al, 2003). More recent data suggests that these effects may be mediated through the regulation of claudin 2, a tight junction protein responsive to IL-6, whose upregulation reduces the integrity of the epithelial barrier (Suzuki et al, 2011). The observations that both IL-6 and claudin 2 may be modulated in both human and murine inflammatory disease of the intestine suggest that this relationship could be of importance in the pathogenesis of inflammatory bowel disease.

Hence, this current data suggests that functionalized dendrimer molecules with the capacity to influence mucosal integrity in the gut can be generated in a scaleable, high purity and cost-effective manner. While the glycosylated dendrimers in this study are targeted at MD-2 binding by Lipid A, these data also suggest the possibility that functionalized dendrimer-based constructs with differing selectivity could be targeted for gastrointestinal bioactivity. This study raises the intriguing possibility that such dendrimers may have a clinical utility in the management of the mucosal damage produced by both gastrointestinal infection and inflammation.
